# Basket Trials for Intractable Cancer

**DOI:** 10.3389/fonc.2019.00229

**Published:** 2019-04-12

**Authors:** Bao-Dong Qin, Xiao-Dong Jiao, Ke Liu, Ying Wu, Xi He, Jun Liu, Wen-Xing Qin, Zhan Wang, Yuan-Sheng Zang

**Affiliations:** Department of Medical Oncology, Changzheng Hospital, Naval Medical University, Shanghai, China

**Keywords:** basket trial, intractable cancer, molecular alteration, personalized precision therapy, genome-driven oncology, refractory cancer

## Abstract

Currently, genomic characterization has become standard of care for tumor types such as non-small cell lung cancer, breast cancer, melanoma, and colorectal cancer. A deep understanding of genomic alterations in different tumor types would help identify potentially actionable genomic changes which occur across a wide variety of tumor types. A basket trial is a new type of clinical trial for which eligibility is based on the presence of a specific genomic alteration, irrespective of histology. Basket trials are phase II screening trials for the off-label use of a targeted drug in patients with the same genomic alterations for which it was approved. Intractable cancer refers to a type or condition of cancer which is unresponsive or resistant to treatment; intractable cancers may be classified into five subtypes as follows: hard-to-treat condition of common advanced cancer after multiple-line therapy, rare cancer in which no standard of care has been recommended, advanced cancer in which standard of care does not work well, cancer accompanied with organ dysfunction, and cancers in older or younger cancer patients. Previous studies have demonstrated that in basket trials, genomic-guided therapy yields clinical benefits in intractable cancer, thereby providing novel insights into the optimal clinical management of such cancers. In this review, we describe a novel way to classify intractable cancer, and summarize the current knowledge on such cancers. We additionally provide information on the role of basket trials in intractable cancer.

The landscape of genome-driven oncology was established based on the understanding of the detailed genetic profiles of tumors and attempts to target gene alterations. Advancements in sequencing technologies as well as innovations in the development of drugs that target molecular alterations have brought forth the promise of genome-driven oncology care (1). Basket trials have been formulated to investigate the efficacy of molecular-targeted therapy for oncogene-defined subsets of cancers across different tumor histologies (2). While basket trials might be an useful design regardless of the setting, the use of basket trials might be especially useful when the cancer is intractable (3, 4). Therefore, we will provide a review of intractable cancer settings with some specific examples to illustrate the need for improved therapy in these cases.

## Intractable Cancer

**Figure 1:**
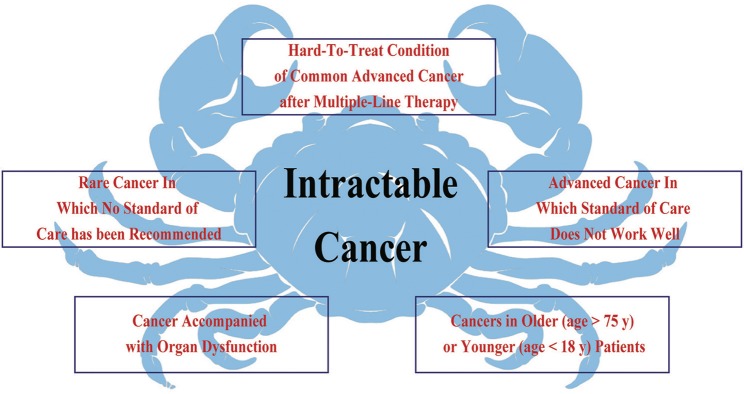
Intractable cancer refers to a type of cancer or condition of cancer that does not respond to/is resistant to cancer treatment. The common characteristics include, no standard treatment for that cancer type and status, standard treatment that has not worked well, and lack of highly effective and low-toxicity regimens. Therefore, intractable cancer could be divided into five subtypes.

Intractable cancer refers to a “hard-to-treat” cancer or condition of cancer that does not respond to/is resistant to cancer treatment, or for which standard of care treatment has not been defined. Common characteristics of intractable cancer include: (1) no standard treatment for that cancer type or condition; (2) low response to standard treatment; and (3) lack of highly effective and low-toxicity regimens. Based on these characteristics, we classified intractable cancers into the following five subtypes (Figure 1): (a) hard-to-treat condition of common advanced cancer after multiple-line therapy; (b) rare cancer in which no standard of care has been recommended; (c) advanced cancer in which standard of care does not work well; (d) cancer accompanied by organ dysfunction; and (e) cancers in older (>75 years) or younger (<18 years) patients.

### Hard-to-Treat Condition of Common Advanced Cancer After Multiple-Line Therapy

Hard-to-treat conditions in patients with common advanced solid tumors who have experienced failure of multi-line standard treatment are a common subtype of intractable cancer. Advancements in modern oncology treatments have significantly prolonged disease control and consequently improved quality of life of patients. Patients who fail standard treatment often have a good performance status, and can be offered additional lines of treatment. In addition, due to the availability of targeted therapies that are highly effective with low toxicity, patients with advanced solid tumors often have the chance to receive third- and further-line therapy. However, lack of appropriate treatment regimens affects the quality of care, even in patients with a good performance status. The paucity of approved agents for third-line therapy and beyond for patients with advanced cancer constitutes an important unmet medical need. There are a number of well-documented treatment options for first- and second-line therapy for common cancers, but there are less data on the beneficial effects of third-line systemic therapy. Following the second chemotherapy regimen, the likelihood of an objective tumor response and disease control generally decreases with each subsequent line of chemotherapy. For example, the overall response rate (ORR) of docetaxel regimens as first- or second-line therapy was 20.9 and 16.3%, respectively, but was only 2.3% for third-line or further-line therapy in non-small cell lung cancer (NSCLC) (5).

An increasing number of clinical studies have attempted to confirm the effects of chemotherapy in patients who are heavily treated, but are still in good clinical condition. However, the survival advantages are no longer present because of drug resistance and/or a lack of agents with any real efficacy. For example, the objective response rate (ORR) for third-line treatment in lung cancer was only ~6–23% (6). The ORRs for the most common chemotherapy regimens used in third-line therapy for metastatic colorectal cancer vary from 5 to 36% (7).

Due to the low efficacy of chemotherapy in further-line treatment, targeted agent therapies have been considered as ideal regimens with greater efficacy and less toxicity than traditional non-targeted cytotoxic drugs such as anlotinib for NSCLC and regorafenib for colorectal cancer. In the ALTER 0303 trial, anlotinib as third-line therapy or further treatment prolonged the median overall survival (mOS) by only 3.3 months with an elevated ORR (9.2 vs. 0.7%) compared to the placebo group in patients with advanced NSCLC (8). Similarly, in the CORRECT trial, regorafenib prolonged the mOS by only 1.4 months (6.4 vs. 5.0 months) and the median progression-free survival (PFS) by 0.2 months (1.9 vs. 1.7 months), with an elevated ORR of 0.6% (1.0 vs. 0.4%) in metastatic colorectal cancer after standard therapies (9). Anlotinib or regorafenib now constitute the recommended standard for third-line therapies even with the small improvement in prognosis associated with their use; this further confirms the intractability of this subtype and the high demand for novel treatment strategies.

### Rare Cancer in Which No Standard of Care Has Been Recommended

Rare tumors refer to those with an incidence of 6–15 per 100,000 cases per year (10). Epidemiological data have estimated that all subtypes of rare cancers account for 22–25% of all adult tumors, although they are individually uncommon (6, 11). In the United States, rare cancers are believed to be the fourth leading cause of cancer-related death each year, contributing to 25% of cancer mortality cases (12). These numbers are expected to rise as genomic-based classification becomes more prevalent, resulting in the increased identification of rarer, molecularly-defined subgroups of cancer (12). Hence, the overall burden of rare tumors is significant. For example, rare cancers accounted for 24% of all cancers diagnosed in Europe during 2000 to 2007, with an annual rise in incidence of 0.5%. The 5-year relative survival rate for all rare cancers is 48.5% compared to 63.4% for common cancers, only increasing by 2.9% from 1999–2001 to 2007–2009 (13). Rare cancers pose challenges for diagnosis, treatments, and clinical decision making (14).

The core challenge and difficulty may be the rarity of such cancer types. Clinical management of rare malignancies is challenging due to lack of information about diagnosis as well as a shortage of approved therapeutic options (15). Rare cancers are also scientifically challenging to study, because most reports on these cancer types tend to be case studies rather than phase III clinical trials (16). Thus, there is a paucity of standard recommended regimens with supporting high-quality evidence for the treatment of rare cancers. Notably, advances in cancer biology and genomic technology have also led to the in-depth understanding of the biology of rare tumors. For example, a previous study reported that 92.5% of rare patients had ≥1 actionable target, and that the outcome could be improved when the patient received matched targeted therapy (17). This customized precision strategy has provided insights into the development of novel treatment regimens for rare cancers. While clinical decision-making in rare cancers has historically been difficult due to the low numbers of cases, new treatment strategies are currently being explored due to improved identification of these rarer entities; flexibility and innovation in clinical trial design has also allowed for testing of multiple drugs in multiple rare cancer patients.

### Advanced Cancer in Which Standard of Care Does Not Work Well

In clinical practice, for several cancer types, recommended standard of care has been established, but these standard regimens do not work well or elicit a week response; examples of such cancers include hepatocellular carcinoma (HCC), biliary tract cancer, pancreatic cancer, and large-cell neuroendocrine carcinoma. Thus, these cancers are considered hard-to-treat due to the lack of highly effective regimens. Sorafenib is an effective first-line treatment for unresectable HCC, and leads to disease stabilization which can prolong OS by 2–3 months; however, the ORRs are very low at 2–5% (18, 19). Targeted therapy for HCC in the past 10 years has been marked by several failed global phase III trials which did not show non-inferiority or superiority to sorafenib (20–23). Before the REFLECT study, no approved first-line systemic treatments were available for advanced HCC other than sorafenib, although sorafenib treatment was associated with a low ORR. The REFLECT study is the first trial to show positive results for sorafenib therapy in HCC (24). Although lenvatinib has shown statistically significant clinically meaningful improvement in PFS (7.3 months) or ORR (24.1%) for HCC treatment, its efficacy as first-line treatment is not satisfactory. With the emergence of innovative molecular-targeted agents and novel therapeutic strategies (e.g., immunotherapy), a combination of different targeted agents or immunotherapy has been a popular topic of research (25).

With the exception of HCC, biliary tract cancer is another intractable cancer type in which first-line treatment does not work well. Two randomized trials (ABC-02 and BT22) provided supporting evidence for the use of gemcitabine combined with cisplatin as standard first-line treatment for biliary tract cancer, but the effectiveness of this regimen needs to be enhanced in the first-line setting (26–28). The median OS of standard first-line therapy is modestly approaching 1 year with an ORR of 19.5–26.1%; however, almost 70% of patients who receive this regimen develop grade 3 or 4 toxicity. This standard of care is representative of a regimen with low efficacy and high toxicity. In addition, no other established standard regimens in the second-line setting are available when gemcitabine regimens fail (29). Standard treatments for pancreatic cancer and large-cell neuroendocrine carcinoma also have low efficacy (30, 31).

### Cancer Accompanied With Organ Dysfunction

Organ dysfunction in cancer patients is a common occurrence, and is associated with an increased risk of mortality. A recent study found that hyperlactacidemia, number of dysfunctional organs, and liver dysfunction were independent risk factors for mortality (32). Thus, comprehensive support therapy is vital to improve this type of intractable cancer. On the other hand, organ dysfunction (especially hepatic and renal dysfunction) affects the metabolism and excretion of anti-tumor agents, further influencing the efficacy and/or safety of such agents in cancer patients; this often leads to dose reductions. However, most dose adjustments are empiric, and balancing the adjustment of certain anti-tumor agents to prevent excessive toxicity with the risk of undertreating the disease remains a concern (33). Meanwhile, the recommendations for dosing adjustments are based on studies in small cohorts or on case studies without high-quality evidence (34, 35). Therefore, personalized dose adjustments and regimens with low organ toxicity may improve efficacy and safety of treatments for these hard-to-treat intractable tumors.

### Cancers in Older (Age > 75 Years) or Younger (Age < 18 Years) Patients

In the coming years, the number of elderly patients with cancer will considerably increase, for example, the incidence of cancer is 11-fold higher after the age of 65 years compared to that at 65 years of age and younger (36). However, despite the high incidence of cancer in older patients, administering treatment is challenging. Most elderly cancer patients (age >75 years) have been excluded from clinical trials because of age limits or comorbidities. A recent survey from 25 European Organization for Research and Treatment of Cancer randomized trials involving more than 6,024 patients only included 9% of patients aged 70 years or older (37). Therefore, solid data regarding the most appropriate approach and optimal treatment for older cancer patients are lacking due to their underrepresentation in prospective clinical trials. The results of several studies analyzing treatment in the elderly have revealed the overall diminished use of chemotherapy despite significant benefits for those who can receive therapy with equal or at least manageable toxicity (38–40). These data suggest that elderly patients may be a vulnerable population, and as such, anti-tumor therapy should sometimes be withheld in such populations. In fact, the decision-making process is much more complicated for elderly cancer patients. Older cancer patients are highly heterogeneous in all domains of physical and psychological function; thus, to treat hard-to-treat cancers in this group, we need a higher participation of elderly patients in clinical trials and more applicable data pertaining to patient characteristics, treatment responsiveness, and the toxicity profile in such patients. The most important outcomes of treatment in elderly cancer patients, namely PFS, time to treatment failure, and even OS, may be less important than the preservation of independence, good performance status, and quality of life (41, 42). Less toxic and personalized therapy should be considered for this type of intractable cancer.

Some organs and body systems can still be growing and developing in younger cancer patients, which can make such patients more sensitive to treatments such as chemotherapy and radiation therapy. However, specific tumor characteristics as well as the diversity of physical and psychological functions render younger cancer patients “hard-to-treat.” Cancer in younger patients (age <18 years) can be classified into two subtypes. One subtype is solid tumors in younger patients which are also common in adults, such as breast and colorectal cancers. In these cases, similar to elderly cancer patients, most therapeutic trials in oncology do not admit younger cancer patients. These younger patients are more likely to receive treatments recommended for adults. Although younger patients are generally better able to recover from higher doses of chemotherapy than are adult patients, there is no standard of care. Meanwhile, failure after multiple-line therapy makes these patients “hard-to-treat,” similar to adult patients. The other subtype is cancer that is common only in younger patients, such as soft tissue sarcoma (STS). The relative rarity of cancer among younger patients limits the familiarity of oncologists with the diagnostic and therapeutic aspects (43). For example, STS is a biologically heterogeneous malignancy with more than 50 subtypes. Traditional cytotoxic agents have limited clinical benefit beyond the first-line setting. Across all high-grade STS subtypes, median OS remains ~12–18 months for advanced metastatic disease (43). After resistance to first-line regimens, highly effective second-line agents which can be used in younger cancer patients are lacking. Further progress in the management of this type of intractable cancer will rely on novel trial design, subtype-specific therapies, and validation of biomarkers to tailor therapy.

## Basket Trials

**Figure 2:**
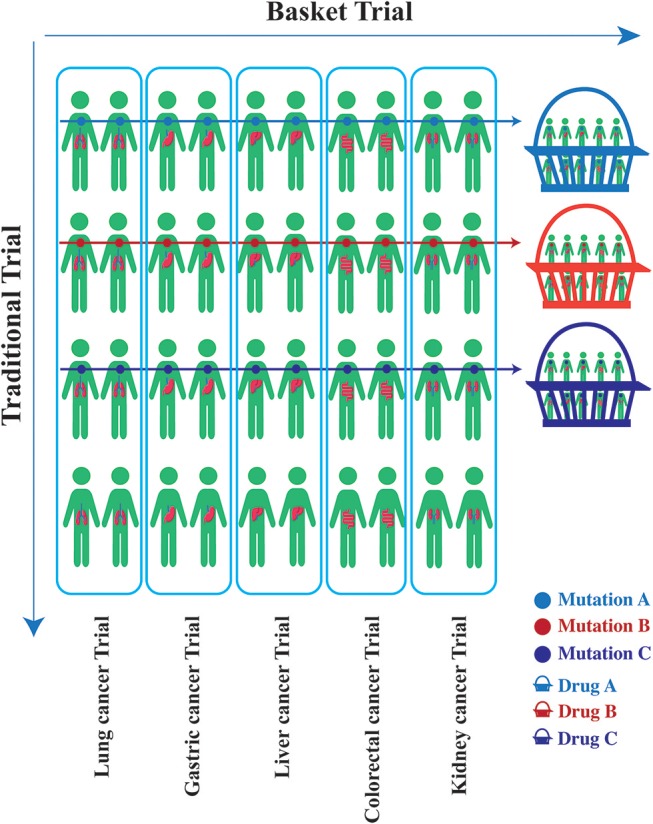
Unlike traditional clinical trials which focus on patients with a single cancer histology, the core organizing principle of basket trials is concentrated on a specific genomic alteration found in the tumor, regardless of where the cancer originated. A basket trial tests the effect of one drug (Drug A/B/C) that targets the same genomic alteration (Mutation A/B/C) across a variety of tumor types.

The initial proof-of-concept of genome-driven oncology was the development of imatinib for chronic myelogenous leukemia in patients harboring the BCR-ABL translocation (44). Subsequently, agents targeting human epidermal growth factor receptor 2 (HER-2)-overexpressing breast or gastric cancer, BRAF V600E-mutation melanoma, and EGFR-, ALK-, and ROS1-mutation lung cancer enables patients with those molecular alterations to have a significantly prolonged life expectancy. These early successes in identifying and targeting cancer-driving genomic alterations have propelled a paradigm of choosing therapy strategies guided by an individual tumor's genomic profile (45). Genomic characterization has become the standard of care for some cancer types such as lung cancer, breast cancer, melanoma, and colorectal cancer. With the deep understanding of genomic alterations in different tumor types, recognition that potentially actionable genomic changes could occur across a wide variety of tumor types have been proposed, although with low frequency in any individual tumor type (46). Could drugs that target specific gene alterations cause responses across a wide variety of diseases? Could drugs work based on genomics rather than the site of origin of the cancer? To address these questions, a novel clinical design called a “basket trial” was developed. This is a new clinical trial paradigm that determines eligibility based on the presence of a specific genomic alteration, irrespective of histology (1, 47, 48). Unlike traditional clinical trials which focus on patients with a single cancer histology, the core organizing principle of basket trials focused on a specific genomic alteration found in the tumor, regardless of where the cancer originated (Figure 2). Basket trial is virtually phase II screening trial for the off-label use of a targeted drug in patients with the same genomic alterations, and there are several potential advantages in the precision oncology era.

First, a basket trial may provide initial proof-of-principle evidence for the clinical validation of a newly discovered tumor-driven target, especially for uncommon, low frequency, or orphan genomic alterations. Since all participating patients share a genomic alteration that drives cancer progression across tumor types, a high sensitivity to targeted therapy is theoretically possible. In addition, the low incidence of these genomic segments makes randomized trials challenging, and thus basket trials are optimal for investigating the efficacy of target therapies. NTRK (NTRK-1, 2, 3) translocations have been observed in <1% of cancers and in more than 20 cancer types. Drilon et al. used a non-comparative, genomic-driven, single-arm basket trial in 55 patients with TRK translocations across 12 different tumor types, and found that the ORR of larotrectinib (LOXO-101, a TRK inhibitor) was 80%, and 71% of the responses were still ongoing at 1 year (49). This study has made a significant impact on clinical practice in cancer patients harboring the TRK translocation, and also illustrates the potential for future advancements in drug development and clinical trials of cancers harboring rare genomic alterations (50).

Second, a basket trial may allow screening for potential efficacy across multiple tumor types in order to guide more traditional, disease-specific, follow-up studies. The goal of some basket trials is to obtain an overall assessment of the drug with pooled histologies; in such trials, subsequent analyses are straightforward, and are comparable to those in standard phase II trials. Some basket trials aim to evaluate the possibility that the efficacy of targeted therapy depends on the histology; several such trials have been unable to find evidence of trans-tumor efficacy of the targeted therapy being studied. In these cases, the targeted drug regimen can be evaluated separately for each histology by increasing the sample size to conduct separate two-stage phase II trials for each histological type (48). If the ORRs appear heterogeneous, a two-stage phase II design is conducted separately for each histology. In the basket trial of vemerafinib in patients with BRAF V600E mutations, the drug was active in NSCLC and several other histologies, but not in colorectal cancer. Therefore, follow-up studies were conducted separately in the NSCLC cohort.

Third, a basket trial can determine if new drugs effectively inhibit their intended targets, by evaluating them in optimal candidates based on genomic selection. In drug development, pre-clinical trials assess the inhibition of targets both *in vitro* and *in vivo*. After achieving satisfactory results on safety and toxicity, the inhibitory activity of targeted agents in tumor patients can be tested. Basket trials can identify patients with identical mutations across different tumor types to achieve clinical confirmation. For example, the STARTRK-2 study is a basket study that assessed the efficacy of RXDX-101 (entrectinib) for the treatment of patients with solid tumors harboring the NTRK1/2/3, ROS1, or ALK gene fusions. A total of 54 patients carrying the NTRK gene fusion had an ORR of 57.4% with a median OS of 20.9 months, and 32 patients with ROS-1 fusion had an ORR of 78% with a median PFS of 29.6 months, suggesting that entrectinib exhibits highly effective NTRK or ROS1 inhibitory activity (51).

Fourth, a basket trial can generate pivotal data that support new standards of care. In clinical practice, some cancer types such as Erdheim-Chester disease and Langerhans cell histiocytosis (LCH) lack a recommended standard of care. In the basket trial of vemurafenib in patients with BRAF V600E mutations, patients with Erdheim-Chester disease or LCH had significant clinical benefits from vemurafenib (52). Thus, this basket trial established a standard of care for these rare cancers with BRAF mutation. Another example is basket trial testing of programmed cell death protein 1 (PD-1) blockade in patients with deficient mismatch repair (dMMR)/microsatellite instability-high (MSI-H) tumors, which confirmed that dMMR can predict the response of solid tumors to PD-1 blockade; this trial has led to the regulatory approval of pembrolizumab for dMMR patients, regardless of cancer origin (53). It generated a novel standard of care for cancer patients with dMMR/MSI-H.

## The Role of Basket Trials in Intractable Cancer

Initially, the critical use of a basket trial is to determine whether a drug already approved to target a genomic alteration in one cancer type is efficacious against the identical mutation in other cancer types. Theoretically, a druggable genomic alteration is also vital to the initiation and progression of cancer, and drugs targeting tumor-driving alterations could block cancer cell metastasis, providing clinical benefits to patients with intractable cancer. Given the increasing number of drugs that target genomic alterations in a tumor-specific context and the rapid development and popularity of tumor genome sequencing, the potential for broadening the utility of genome-driven oncology though basket trials has been explored in intractable cancer. To achieve the treatment value of basket trials in intractable cancer, two questions must be answered: (a) do these intractable cancer patients harbor a druggable molecular alteration; and (b) is targeting specific alterations still efficacious in intractable cancer patients carrying identical mutations.

### Frequency of Druggable Molecular Alterations in Intractable Cancer

Genome-driven oncology has helped establish the effectiveness of the application of clinical genomics to treatment (54). In certain cancers like those of the lung, it has become standard practice to profile tumors for targetable mutations. The application of next-generation sequencing (NGS) has enabled the marked expansion of molecular profiling in cancer patients. In 2017, the Memorial Sloan Kettering Cancer Center (MSKCC) revealed the mutational landscape of metastatic cancer using the MSK-IMPACT NGS panel in 10,336 patients with advanced disease; these patients were frequently heavily treated. The data included details of more than 300 tumor types. The results revealed that a total of 36.7% of patients (*N* = 3792) harbored at least one actionable alteration (55). Many druggable targets were shared across these patients with intractable cancer (Table 1).

**Table 1:** The frequency of common molecular alterations in pan-cancer based on the data from MSK-IMPACT sequencing(%).

	***N***	**EGFR** **mutation**	**EGFR** **amplification**	**ROS1** **fusion**	**BRAF** **mutation**	**Her-2** **amplification**	**Her-2** **mutation**	**PIK3CA** **mutation**	**CDK4** **amplification**	**CDK6** **amplification**	**Met** **amplification**	**BRCA1/2** **mutation**
NSCLC	1, 668	18.65	1.68	1.62	5.16	21.7	3.23	6.89	3.84	0.3	2.1	5.22
Breast cancer	1, 324	1.36	1.44	0	0.3	13.22	3.63	34.59	1.44	0.3	0.15	5.29
Colorectal cancer	1, 007	2.09	1.29	0	11.52	2.38	3.87	19.17	0	0.3	1.19	8.54
Prostate	717	0.56	0.42	0	1.67	0	0.98	3.35	0.7	0.42	0.14	5.58
Pancreatic cancer	502	0.8	0.2	0.2	2.39	1.2	0.6	3.39	0	0.8	0	3.07
Soft tissue sarcoma	443	0.9	0.9	0.45	1.35	0	0.68	3.84	17.16	0.68	1.35	2.15
Bladder cancer	423	4.49	1.65	0.24	3.78	4.49	10.87	22.93	1.42	0.24	0.24	14.66
Melanoma	365	6.58	0.55	0	30.41	1.1	3.01	4.11	3.29	0.82	1.64	12.6
Renal cell carcinoma	361	1.11	0	0	0	0	1.66	3.88	0	0	1.66	1.11
Hepatobiliary cancer	355	1.13	1.41	0	3.1	2.54	0.56	3.94	0.85	1.13	0.56	2.25
Esophagogastric cancer	341	0	0	0.29	1.47	0	0	7.04	1.17	3.81	2.64	5.28
Thyroid cancer	231	0	0	0	38.1	0	0.43	6.06	0.43	0	0	1.73
Ovarian cancer	224	0.45	0	0.89	1.34	3.57	1.34	10.27	0.89	0	0.89	3.13
Endometrial cancer	218	4.13	0	0	4.13	7.34	4.59	40.37	0.46	0	0	7.63
Head and neck caner	186	2.15	6.99	0	0.54	2.69	1.08	19.35	0.54	1.61	0	5.38
Cancer of unknown primary	186	3.23	2.69	0.54	5.91	4.3	3.76	9.14	1.61	0.54	0.54	5.46

*http://cbioportal.org/msk-impact*.

ProfiLER is another molecular profiling clinical trial that explored cancer cell genomic alterations in 2,676 patients with 16 refractory cancer types to guide treatment. A total of 1004/1944 (52%) patients had at least one actionable mutation: 609 patients had one actionable mutation, and 394 had two or more actionable mutations. Molecular-target therapy was also recommended in 676/1944 (35%) patients with refractory cancer (56). Similarly, the SHIVA study assessed the off-label use of molecularly targeted agents in 741 patients with metastatic solid tumors refractory to the standard of care, and found that 293 (40%) refractory cancer patients had at least one druggable molecular alteration (57). Besides, the MOSCATO-01 study demonstrated that an actionable molecular alteration could be identified in 411/843 (49%) patients with advanced hard-to-treat cancers (58). This was also the first trial to investigate characterized genomic alterations in recurrent or refractory solid tumors in pediatric patients (59). Successful molecular analyses in 69 pediatric patients revealed that 60.9% (42/69) of patients have actionable alterations in various oncogenic pathways. Among these, 26% had more than one actionable alteration, 42.4% harbored a target detected by copy-number analysis, 33.3% had an actionable mutation, and 14.5% had both. With regard to rare cancers, Kato et al. reported 37 patients (92.5%) had at least one potentially actionable target among 40 patients who received genomic and protein analyses (17).

Based on the data from these studies, it is evident that the frequency of druggable molecular alterations in intractable cancer is not low; this allows clinicians or physicians to conduct genomically-guided clinical trials to evaluate the efficacy of approved and investigational molecularly targeted therapies across distinct tumor types with shared genetic features in those with intractable cancer.

### Effects of Targeted Therapy on Intractable Cancers Carrying Identical Mutations

Previous studies have provided validation for the use of genomics in clinical practice, and have shown that it could very well guide treatment choices for patients with intractable cancer (Table 2). In 2010, Von Hoff conducted a pilot study using molecular profiling to identify potential targets and select treatments for refractory cancers (67). A molecular target was detected in 84 of 86 patients with refractory metastatic cancer, and 18 of 66 patients (27%) had a longer PFS (67). In this trial, Von Hoff also proposed an endpoint called PFS2/PFS1 (PFS on genomic-guided targeted therapy/PFS on prior therapy), and concluded that a ratio > 1.3 would indicate a treatment benefit (67); such a benefit is often considered a clinical benefit in basket trials.

**Table 2:** Published and ongoing basket trials for intractable cancer.

**Study**	**Tumor type**	**Country/region**	**Genetic targets**	**Number**	**Clinicaltrials.gov Identifier**
**PUBLISHED TRIALS**					
ProfiLER (56)	Solid tumor	France	KRAS, PIK3CA, CDKN2A, CCND1, FGFR1, MDM2, HER2,HER1 EGFR, VEGF, CDK, etc	2676	NCT01774409
SHIVA (57, 60)	Solid tumor	France	PI3K/AKT/mTOR, RAS/RAF/MEK	741	NCT01771458.
STARTRK-2 (51)	Solid tumor	United States	NTRK1/2/3, ROS1, ALK	300	NCT02568267
MyPathway (4, 61)	Solid tumor	United States	HER2, BRAF, EGFR, Hedgehog	251	NCT02091141
MOSCATO-01 (58, 59)	Solid tumor	France	PIK3CA, HER2, PTEN, FGFR1, EGFR, NOTCH, RAS, MET, FGF, RB1, RAF, CDK, MDM2, etc	199	NCT01566019
VE-BASKET (52, 62–64)	Solid tumor Multiple myeloma	United States	BRAF	208	NCT01524978
15-335 (65)	Lung cancer; Bladder cancer; Urinary tract cancer;	United States	HER2	100	NCT02675829
Hasegawa K's study (66)	Gynecologic cancer	Japan	PIK3CA	20	Japic CTI-132287
**ONGOING TRIALS**					
Lung-MAP	Squamous cell lung carcinoma	United States	FGFR, CDK4/CDK6/CDKN2A, PI3K, etc.	10000	NCT02154490
TAPUR	Solid tumor; Multiple myeloma; Non-hodgkin lymphoma	United States	ALK/ROS1/MET, CDKN2A/CDK4/CDK6, mTOR/TSC, HER2, BRAF, RAS, RET, VEGFR, KIT, BRCA1/2, POLE/POLD1, MSIH, etc	2980	NCT02693535
NCI-MATCH	Solid tumor; lymphomas; myeloma	United States	EGFR, MET, ALK, ROS1, HER2, FGFR, mTOR, TSC1/2,GNAQ/GNA11, SMO/PTCH1, c-KIT, CDK4/6, NTRK, PIK3CA, PTEN	3000	NS
SAFIR02-Breast	Breast cancer	France	mTOR,EGFR, AKT, MEK, Her2,VEGF, PARP, AR, etc	1460	NCT02299999
MOSCATO-02	Solid tumor	France	NS	1050	NCT01566019
SAFIR02-Lung	NSCLC	France	mTOR,FGFR, AKT, MEK, Her2,VEGF, PARP, etc	993	NCT02117167
CUPISCO	Cancer of unknown primary site	United Kingdom	ALK/ROS1/MET, BRCA1/2, EGFR,VEGFR, BRAF, Hedgehog, AKT, MEK, HER2, etc.	790	NCT03498521
RNASARC	Soft tissue sarcoma	France	NTRK1/2/3, ROS1, ALK	750	NCT03375437
MAPPYACTS	Pediatric tumor	Europe	NS	700	NCT02613962
CREATE	Rare cancer (PRCC2, ASPS, CCS, ARMS, IMFT, ALCL)	Europe	ALK, MET	582	NCT01524926
ESMART	Solid tumor (age <18 years)	France	BRCA1/2, PI3K/AKT/mTOR, CDK, etc	397	NCT02813135
SHIVA-02	Solid tumor	France	PI3K/AKT/mTOR, RAS/RAF/MEK	370	NCT03084757
HETIAN64	Solid tumor	China	EGFR, HER2, ALK/ROS/MET, BRCA1/2, CDK4/6, mTOR/PI3KCA, RET, BRAF, etc	60	NCT03239015

*PRCC1, Papillary renal cell carcinoma; ASPS, Alveolar soft part sarcoma; CCS, Clear cell sarcoma; ARMS, Alveolar rhabdomyosarcoma; IMFT, Inflammatory myofibroblastic tumor; ALCL, Anaplastic large cell lymphoma; NS, None Stated*.

Subsequently, Hyman et al. conducted one of the most famous basket trials (52). A total of 120 patients with refractory cancer who harbored BRAF mutation received vemurafenib. In NSCLC patients after multiple-line therapy, the ORRs were 42% with a disease control rate (DCR) of 84%. This rate compares favorably with the 7% ORR reported for standard second-line docetaxel in molecularly unselected patients (68, 69). The DCR was 62% in a cholangiocarcinoma cohort, which was also better than second-line chemotherapy (49.5%), although there are no standard second-line regimens for this refractory cancer (29). This study demonstrated that histology-independent, biomarker-selected basket studies are feasible and can serve as tools for developing molecular-targeted cancer therapies for intractable cancer. Meanwhile, multiple basket studies have also been conducted in intractable cancers. The MyPathway study included 251 patients with 35 different tumor types who had advanced refractory solid tumors harboring molecular alterations in HER-2, EGFR, BRAF, and Hedgehog (4). Patients with refractory, metastatic HER2-amplified/overexpressing colorectal cancer accounted for the largest treatment group in this study. A total of 37 colorectal cancer patients with HER-2 amplification/overexpression had an ORR of 38% and a median duration of response of 11 months, which compares favorably to the ORR of other drugs that were recently approved for refractory colorectal cancer. HER-2 targeted therapy is also efficacious in other refractory cancers such as biliary tract cancer (ORR, 29%) and bladder cancer (ORR, 33%). In particular, HER-2 targeted therapy has an ORR of 80% in rare salivary duct carcinomas.

For rare cancers and younger cancer patients, basket trials have also shown improvements in prognosis. For example, imatinib has been used in diverse rare cancers known to express imatinib-sensitive tyrosine kinases. A basket trial showed responses among multiple rare malignancies including dermatofibrosarcoma protuberans, hypereosinophilic syndrome, myeloproliferative disorders, and systemic mastocytosis, which facilitated the FDA approval of imatinib for these rare and ultra-rare disease conditions (70). Furthermore, another basket trial assessed 21 rare cancer patients who received matched therapy for an actionable alteration; 52.4% (11/21) attained stable disease (SD) > 6 months, partial responses (PR), or complete response (CR) (14.3% had SD > 6 months; 28.6% had PR; 9.5% had CR) with a median PFS of 19.6 months. Moreover, the matched therapy approach resulted in a statistically significant improvement in PFS compared with the last prior unmatched therapy (17). Genomic-guided targeted therapy also renders clinical benefits for younger cancer patents. In the pediatric subgroup of the MOSCATO-01 study, 14 pediatric patients with genomic alterations received a “matching” target agent including clinical trial agents and registered agents, and 5 (35.7%) experienced an objective tumor response. This outcome is ideal for pediatric patients who have received median two (ranged from 1 to 8) prior lines of treatment. For other refractory cancers, basket trials in patients with cancers of unknown origin were also conducted. The CUPISCO study is a phase II, randomized, multi-center study that aims to compare the efficacy and safety of molecular-guided therapy vs. standard platinum-containing chemotherapy in patients with cancer of unknown primary site who achieved disease control after three cycles of first-line platinum doublet induction chemotherapy (NCT03498521, Table 2). This trial is currently recruiting patients.

Basket trials have also provided an efficient method for patients with intractable cancer harboring actionable germline alterations. Study 42 is an open-label non-comparative single-arm phase II study that examined olaparib monotherapy in germline BRCA1/2-associated cancers, regardless of tumor type (71). A prolonged tumor response was seen across a spectrum of malignancies. Specifically, 31.3, 12.9, 21.7, and 50.0% of patients with ovarian, breast, pancreatic, and prostate cancers, respectively, who were heavily treated with a mean number of 4.6 prior chemotherapy regimens in the metastatic setting, achieved a response. In this study, the potential efficacy of poly (ADP-ribose) polymerase (PARP) inhibitors in pancreatic, breast, and prostate cancers was preliminarily validated. This was also the first study to support the efficacy of PARP inhibitors in platinum-resistant or platinum-refractory ovarian cancer. Based on data from this basket trial, subsequent studies, such as the SOLO1 and SOLO2 were performed, and revealed marked antitumor activity in patients with advanced ovarian cancer harboring the gBRCA1/2 mutation. The germline status of BRCA1/2 in individuals with cancer defines a target population in whom PARP inhibitors seem beneficial, supporting the hypothesis that therapy directed against a genetically defined target has activity regardless of anatomic organ of origin.

Besides, the MOSCATO-01 and ProfiLER studies also demonstrated that molecular-targeted cancer therapy could bring survival benefit for intractable caner. For example, in the MOSCATO-01 study, 199 patients were treated with a targeted therapy matched to a genomic alteration, and a PFS2/PFS1 ratio > 1.3 was observed in 33% of patients (63/193). Objective responses were observed in 22 of 194 patients, and the mOS was 11.9 months. Also, the ProfiLER study showed that a total of 53.7% of 143 patients who received the recommended targeted therapy were alive at 3 years, compared with 46.1% of 502 patients who did not receive that therapy. The 5-year survival rate was also higher for patients who received targeted therapy (34.8 vs. 28.1%) (56).

However, there have also been some controversial findings regarding the role of basket trials in intractable caner. For example, the SHIVA study is a two-arm control trial which aims to investigate the efficacy of molecular-targeted therapy in refractory cancer. One group received molecularly targeted agents (MTAs) base on tumor molecular profiling, while the other group received treatment based on the physician's choice (TPC). The SHIVA study demonstrated that the use of MTA outside their approved indications did not improve PFS compared with TPC in heavily treated cancer patients. In the MTA group, the median PFS was 2.3 months compared with 2.0 months in the TPC group, without significant statistical difference. Subsequent crossover analyses revealed that the proportion of patients with a PFS ratio >1.3 was 37 and 61% in those who crossed-over from the TPC to MTA, or from MTA to TPC, respectively. Indeed, a substantial proportion of patients could also get clinical benefit from the treatment algorithm evaluated in the SHIVA trial (60).

Whether the use of genomic-guided therapy can improve outcome in intractable cancer patients is still a controversial topic, and there are several evidences which can help resolve this issue. First, targeted agents can affect the results of a basket trial. It is well-established that only therapies that hit the target with high bioactivity can improve outcome. For example, vemurafenib dramatically improves the outcome of BRAFV600-mutant melanoma, whereas sorafenib, a weak BRAF inhibitor, does not have this effect (72). In the MOSCATO trial, 75% of patients received last-generation targeted therapies in the phase I/II trial, and thus, a positive outcome was obtained in this basket trial. Second, extensive molecular analysis can identify specific genomic contexts that may condition the response to targeted therapy. A recent multihistology basket trial of an AKT inhibitor (AZD5363) in advanced solid tumor refractory to standard therapy with AKT1 E17K mutation illustrated the utility of comprehensive tumor biomarker analysis. Overall, the median PFS for these patients who received a median of five lines of prior therapy was 4.2–6.6 months. In exploratory biomarker analyses, imbalance of the AKT1 E17K–mutant allele as well as the presence of coincident phosphatidylinositol-4,5-bisphosphate 3-kinase (PI3K) pathway hotspot mutations was associated with a longer PFS (73). If the outcome determination relied solely on the presence or absence of a single molecular alteration in basket trials, we could overlook additional factors that inform optimal patient selection. Therefore, to better understand the underlying biology of this heterogeneity in response, basket trials should evolve to more routinely incorporate comprehensive genomic analyses. Third, tumor location is important in assessing the outcome. For example, in a recent basket study evaluating the efficacy of a pan-HER inhibition (neratinib) in refractory tumors harboring activating mutations in HER2/3, neratinib activity was influenced by both tumor lineage and mutation type (74). Single-agent neratinib showed activity in breast, biliary, and cervical cancers and not in colorectal and bladder cancers. Missense mutations appeared more sensitive compared with exon 20 insertions. Coincidentally, in the MyPathway study, all seven uterine cancer patients with HER-2 amplification/overexpression had no response to HER-2 targeted therapy. Although a common set of driver mutations exist in each cancer type, the combination of drivers within a cancer type and their distribution within the founding clone and subclones vary for individual patients. This suggests that knowing the clonal architecture of each patient's tumor will be crucial for optimizing treatment (46). Meanwhile, the investigators should be very cautious about over-interpretation of results from basket trial. Especially, there is only little or modest improvement in prognosis. In this setting, basket trials cannot distinguish whether the effect is due to improved prognostic outcomes in patients with the genetic biomarker identified, or due to the treatment itself. Besides, with a basket trial across a number of histologies, and a small number of patients within any one histology, it is possible that variation in outcomes might be due to chance differences, and not due to a true treatment effect. Therefore, the effect of basket trial in intractable cancer required to be further confirmed in the next-step.

## Perspective

In the era of genome-driven oncology, understanding the genomic profile of individual cancer patients has become necessary for delivering a selection of regimens for intractable cancer. In the real-world setting, previous targeted therapy studies have yielded disappointing genomic match rates of about 4%−13% (75). One possible explanation for this is the clinician's limited ability to interpret genomic testing results. The other possibility is that gene panels might not cover the full spectrum of genes, or that gene panels might not adequately detect mutations. Therefore, basket trials for intractable cancer may be conducted efficiently only with an in-depth understanding of annotated genomic data in conjunction with their functional and therapeutic implications. An increasing number of large basket trials have been conducted such as TAPUR and NCI-MATCH (Table 2). Multiple basket trials have assessed whether specific targeted therapies could benefit more patients and have led to more personalized therapies in advanced cancer patients without standard treatment options. We believe that these trials will further illustrate the potential scope of this novel therapeutic strategy. Besides, it remains unclear whether basket trial is feasible in cancer patients with organ dysfunction due to less relevant reports. Theoretically, intractable cancer patients with organ dysfunction could receive this novel therapeutic regimen because targeted therapy had less organ toxicity than chemotherapy. In the future, more and more studies are required to confirm the feasibility of basket trial in intractable cancer patients with organ dysfunction, and this also will be an important direction of basket trial. Basket trials represent an important research tool for the efficient generation of knowledge needed to deliver clinically valuable therapies. Therefore, improvements in the selection of molecular alterations, genomic knowledge-base integration, as well as patient-matching are needed to translate genomic knowledge into clinically meaningful outcomes and to improve the clinical management of these intractable cancers.

## Author Contributions

YW, JL, XH, ZW, and W-XQ were responsible for data analysis, preparation of figures and tables. B-DQ, X-DJ, KL, and Y-SZ made substantial contributions to conception and design of the review, and analysis and interpretation of articles. All authors have been involved in drafting the manuscript or revising it critically for important intellectual content. B-DQ, X-DJ, KL, and Y-SZ have agreed to be accountable for all aspects of the work in ensuring that questions related to the accuracy or integrity of any part of the work are appropriately investigated and resolved.

### Conflict of Interest Statement

The authors declare that the research was conducted in the absence of any commercial or financial relationships that could be construed as a potential conflict of interest.
